# 通过基于活性的蛋白质组分析揭示螺旋霉素衍生物抗肿瘤作用靶点

**DOI:** 10.3724/SP.J.1123.2026.01003

**Published:** 2026-06-08

**Authors:** Renyu YANG, Tingze FENG, Shaojun PEI, Huan QI, Hailong PIAO

**Affiliations:** 1.中国科学院大连化学物理研究所，辽宁 大连 116023; 1. Dalian Institute of Chemical Physics，Chinese Academy of Sciences，Dalian 116023，China; 2.中国科学院大学，北京 100049; 2. University of Chinese Academy of Sciences，Beijing 100049，China

**Keywords:** 螺旋霉素, 活性分子探针, 基于活性的蛋白质组分析, 靶点, 淀粉样前体蛋白, spiramycin, activity-based probe, activity-based protein profiling, target, amyloid precursor protein

## Abstract

可利霉素是一种在医药领域广泛使用的大环内酯类抗生素，主要成分为3种不同的螺旋霉素衍生物。然而随着相关研究的深入，近期发现可利霉素以及多种不同结构的螺旋霉素衍生物都具有一定的抗肿瘤活性，说明利用螺旋霉素作为母核，进行不同的取代基修饰，可能会得到更好的抗肿瘤活性药物。虽然螺旋霉素有着成为抗肿瘤药物的潜力，但是关于其作用靶点及完整机制的研究依然欠缺。为了揭示其抗肿瘤作用机制，本研究采用了基于活性的蛋白质组分析（ABPP）策略，设计并合成了一种高活性螺旋霉素衍生物正己酰螺旋霉素（h-SPM），进而依据其结构合成了一种结构相似且带有适用于ABPP实验官能团的活性分子探针。利用该探针与细胞蛋白质共孵育，使两者结合，从而捕获h-SPM潜在的药物靶点，随后对探针结合的蛋白质进行分离纯化与质谱分析，获得详细的靶点蛋白质信息。进一步通过基因本体（GO）分析挖掘这些蛋白质的性质和功能，得到明确的功能信息，并据此筛选出若干潜在靶点蛋白质。通过上述方法，从蛋白质谱的结果中鉴定出包括淀粉样前体蛋白（APP）、低密度脂蛋白受体（LDLR）在内的多个h-SPM潜在作用靶点，并采用免疫印迹（Western Blotting）方法确定这些蛋白质对h-SPM产生了响应。随后，通过短发夹RNA（shRNA）介导的蛋白质敲低及细胞染色等实验，证明蛋白质APP在该药物发挥作用的过程中扮演了关键角色，初步揭示了该类药物的抗肿瘤作用机制。本研究不仅建立了适用于螺旋霉素类化合物的靶点筛选方法，也为该类药物的后续开发提供了关键靶点线索与理论依据。

## Abstract (en)

Carrimycin is a macrolide antibiotic widely used in the pharmaceutical and clinical fields， with its primary composition consisting of three distinct derivatives of spiramycin. For decades， macrolides like carrimycin have been valued for their efficacy against Gram-positive bacteria and certain atypical pathogens. However， as research has progressed， recent studies have revealed that carrimycin， along with various other structurally modified spiramycin derivatives， exhibits significant antitumor activity in multiple experimental models. This finding suggests that the spiramycin molecular scaffold possesses intrinsic potential beyond its conventional antibacterial role. By strategically modifying the substituents attached to this core structure， it may be feasible to develop novel compounds with enhanced and more selective antitumor properties. Despite this promising outlook， research into the specific targets and comprehensive mechanisms underlying the antitumor effects of spiramycin derivatives remains notably insufficient. A major gap exists in the precise identification of their molecular targets within cancer cells and the detailed signaling pathways they modulate. This lack of mechanistic understanding poses a substantial barrier to the rational design， optimization， and clinical translation of spiramycin-based antitumor therapeutics. To systematically address this knowledge gap and elucidate the antitumor mechanism of this compound class， the present study adopted an activity-based protein profiling （ABPP） strategy. ABPP is a chemoproteomic approach that enables the direct identification of functionally active proteins that interact with small-molecule probes in a native biological context. As a first step， we designed and synthesized a novel， high-activity spiramycin derivative termed *n*-hexanoyl spiramycin （h-SPM）. Building upon the structure of h-SPM， we subsequently engineered and synthesized a structurally analogous activity-based probe. This probe was specifically functionalized with chemical handles （such as an alkyne group） compatible with ABPP methodologies， allowing for downstream bioorthogonal conjugation and enrichment steps. The experimental workflow began by incubating this active probe with live cancer cells. During this co-incubation period， the probe engaged with and covalently bound to its potential protein targets within the complex cellular environment. Following the interaction， cells were lysed， and the probe-labeled proteins were efficiently isolated and purified using affinity-based enrichment techniques—specifically via copper-catalyzed azide-alkyne cycloaddition （click chemistry） to immobilize them onto a solid support. The enriched protein pool was then subjected to in-depth analysis using liquid chromatography-mass spectrometry （LC-MS）. This analytical phase yielded detailed， proteome-wide information on the identities of proteins that interact with the h-SPM-based probe. To extract biological insights from the list of identified proteins， we performed comprehensive bioinformatic analysis using Gene Ontology （GO） enrichment. This systematic classification provided crucial information regarding the biological processes， molecular functions， and cellular components associated with the captured proteins. The functional annotations derived from GO analysis allowed us to evaluate and prioritize several promising candidate target proteins for further experimental validation. Through this integrated proteomic and bioinformatic approach， we successfully identified multiple potential cellular targets of h-SPM. Notable among these were amyloid precursor protein （APP） and low-density lipoprotein receptor （LDLR）， both of which are implicated in diverse cellular processes such as cell adhesion， signal transduction， and metabolic regulation. To confirm the biological relevance of these interactions， we employed Western Blotting experiments. These studies verified that the expression or post-translational modification states of APP and LDLR were altered in response to h-SPM treatment， thereby confirming their status as responsive molecular targets. After identifying and preliminarily validating APP as a key interactor， we proceeded to investigate its functional role in the drug’s mechanism of action. Using short hairpin RNA （shRNA）-mediated protein knockdown， we generated cancer cell lines with significantly reduced APP expression. Comparative analysis of drug sensitivity between these knockdown cells and their wild-type counterparts revealed a marked attenuation of h-SPM’s antitumor effects in the absence of APP. Complementary to this， cell staining assays， including immunofluorescence， were conducted to visualize morphological changes， alterations in cell viability， and the subcellular localization of relevant biomarkers following h-SPM treatment. Collectively， these functional experiments provided compelling evidence that APP plays a critical and indispensable role in mediating the antitumor activity of h-SPM. Our findings thus outline a preliminary model of the drug’s mechanism， likely involving cellular pathways regulated or influenced by APP. In summary， this study achieves two significant objectives. First， it establishes and validates a robust， generalizable target-screening platform based on ABPP， specifically tailored for the investigation of spiramycin-class compounds. Second， and more importantly， it delivers novel biological insights by pinpointing specific protein targets such as APP. The identification of these targets provides invaluable mechanistic clues and a solid theoretical foundation for the future development of this family of compounds. This work effectively advances spiramycin derivatives from compounds with observed phenotypic activity toward agents with an emerging mechanistic understanding， thereby paving the way for more targeted drug design and informed combination therapy strategies in oncology. Future studies will focus on delineating the detailed downstream consequences of APP engagement by h-SPM and exploring the therapeutic potential of other identified targets in preclinical models.

可利霉素是由多种螺旋霉素衍生物组成的混合物，其核心成分为3种螺旋霉素衍生物，通常作为抗生素广泛使用于医药领域^［1，2］^。后续研究进一步表明，该药物亦具备一定的抗肿瘤活性^［3］^。现有关于螺旋霉素类药物的抗肿瘤作用研究表明，该类药物对癌细胞表现出较强的细胞毒性，并可在特定浓度下诱导癌细胞发生凋亡^［2，3］^。对上述现象开展深入的靶点与机制研究，不仅有助于推动该类药物的“老药新用”，也能为其后续临床应用提供重要的理论依据。

本研究合成了一种新的螺旋霉素衍生物正己酰螺旋霉素（*n*-hexanoyl spiramycin， h-SPM），采用基于活性的蛋白质组分析（activity-based protein profiling， ABPP）技术，对该药物的抗肿瘤作用靶点及其分子机制进行探讨。ABPP是一种常用的化学生物学策略^［4，5］^，适用于药物未知靶点的鉴定。药物发挥功能的核心机制之一，在于其分子与细胞内的靶标蛋白质发生非共价相互作用，进而影响蛋白质活性，最终引起相应的生理效应^［6，7］^。这一过程及其产生的生物学结果即为药物作用的分子机制，所结合的蛋白质则被视为药物的靶标分子。

为揭示药物的靶点与分子机制，ABPP方法需借助特定的化学工具分子。具体而言，需要合成一种与目标药物结构类似的活性分子探针，该探针须具备二氮丙啶基团与端炔基团^［8，9］^。将探针加入培养基与细胞共孵育后，其可被细胞摄取；由于结构与药物相似，探针能够模拟药物与相应的靶点蛋白质在细胞内进行非共价结合。随后，用波长为350 nm的紫外光照射细胞，使探针与蛋白质发生交联反应，形成稳定的共价连接，该连接在后续蛋白质提取与纯化过程中不被破坏。提取蛋白质后，通过点击化学反应，使带有不同报告基团的叠氮化物与探针末端的炔基共价连接，形成1，2，3-三氮唑结构，从而实现蛋白质的特异性标记^［10］^。根据叠氮化物的报告基团，可对探针结合的蛋白质进行不同形式的检测与富集。例如，若使用叠氮罗丹明，蛋白质将被标记上荧光基团，便于后续荧光成像或检测；若使用叠氮生物素，则可对探针结合的蛋白质进行生物素标记，进而利用链霉亲和素偶联的固相载体对靶标蛋白质进行特异性富集，从而将其从细胞全蛋白质中分离出来。富集得到的蛋白质经酶解处理后，可进行质谱分析，获得探针结合的蛋白质的组成信息。进一步结合生物信息学分析，即可筛选出潜在的药物靶点蛋白质，并初步推断药物在细胞内的作用机制^［11，12］^。

在获得探针结合的蛋白质信息后，可从中选定若干候选蛋白质作为重点研究对象。针对单个蛋白质的功能验证，通常采用的方法是调控该蛋白质在细胞内的表达水平，继而观察相应的细胞表型变化。本实验利用短发夹RNA（short hairpin RNA，shRNA）介导的基因敲低技术，构建目标蛋白质低表达的稳转细胞系，通过对比该细胞系与野生型细胞在药物处理后的敏感性差异，可判断该蛋白质是否参与药物的作用机制。若特定蛋白质敲低后细胞对药物的反应发生显著改变，则提示该蛋白质很可能为药物的作用靶点之一^［13，14］^。基于ABPP筛选结果，本研究将淀粉样前体蛋白（amyloid precursor protein， APP）和低密度脂蛋白受体（low-density lipoprotein receptor， LDLR）等列为h-SPM的潜在靶标蛋白质。进一步通过shRNA介导的基因敲低技术，证实APP在药物发挥抗肿瘤作用过程中扮演关键角色。生物信息学分析显示，这些候选蛋白质多定位于溶酶体。因此，可通过溶酶体染色等技术，观察h-SPM处理后溶酶体形态与功能的变化，从而深入探讨其潜在的作用途径。综合上述方法，可初步构建药物发挥抗肿瘤作用的分子机制框架。

## 1 实验部分

### 1.1 仪器、试剂与材料

Heracell 240i二氧化碳恒温培养箱（美国Thermo Scientific公司），Milli-Q Advantage A10超纯水仪（美国Millipore公司），Chemi-Smart-2000化学发光荧光成像系统（美国Bio-Rad公司），LTQ-Orbitrap Elite质谱仪，配备Dionex UltiMate 3000 RSLCnano系统（美国Thermo Scientific公司），Oasis HLB固相萃取柱（1 mL/30 mg，美国Waters公司）。

人源乳腺癌细胞MDA-MB-231、人源胶质瘤细胞LN229、人源肺癌细胞A549购自美国模式培养物保藏中心（American Type Culture Collection，ATCC），人源肝癌细胞MHCC-97H购自中国科学院细胞库。

DMEM培养基、RPMI1640培养基、青霉素链霉素溶液、Cell Counting Kit-8（CCK8）细胞计数试剂购于大连美仑生物公司；胎牛血清购于美国Gibco公司；二甲基亚砜（dimethyl sulfoxide，DMSO）、无水甲醇、无水乙醇、氯仿、乙腈（ACN）、甲酸（FA）、胰蛋白酶、无水硫酸铜、叠氮生物素、二硫苏糖醇（dithiothreitol， DTT）、碘乙酰胺（iodoacetamide， IAA）、三氟乙酸（trifluoroacetic acid，TFA）、氨苄青霉素购于上海阿拉丁公司；嘌呤霉素购于法国Invivogen公司；二喹啉甲酸（bicinchoninic acid， BCA）蛋白质浓度测定试剂盒、放射免疫沉淀法（radio immunoprecipitation assay， RIPA）裂解液购于上海翊圣生物公司；丙烯酰胺凝胶、聚偏二氟乙烯（polyvinylidene fluoride，PVDF）膜购于德国Millipore公司；三（苄基三唑甲基）胺（tris（benzyltriazolylmethyl）amine， TBTA）、三（2-羧乙基）膦（tris（2-carboxyethyl）phosphine，TCEP）购于美国Cayman公司；链霉亲和素磁珠购于常州天地人和生物公司；磷酸酶抑制剂混合物、蛋白酶抑制剂混合物、ECoRI内切酶、BshTI内切酶、T4连接酶购于德国Sigma-Aldrich公司；磷酸盐缓冲溶液（phosphate buffer saline， PBS）、十二烷基硫酸钠（sodium dodecyl sulfate， SDS）、尿素购于上海生工生物公司；考马斯亮蓝G250染料试剂、蛋白上样缓冲液、Lysotracker溶酶体染料、4%多聚甲醛溶液、辣根过氧化物酶标记山羊抗人IgG（H+L）购于上海碧云天生物公司；APP抗体、组织蛋白酶 D（cathepsin D， CTSD）抗体、转铁蛋白受体（transferrin receptor， TFRC）抗体、胰岛素样生长因子2受体（insulin like growth factor 2 receptor，IGF2R）抗体购于美国Proteintech公司；LDLR抗体、溶酶体关联膜蛋白1（lysosomal-associated membrane protein 1， LAMP1）抗体购于美国Santa-cruz公司。

### 1.2 细胞培养

细胞培养使用DMEM或RPMI1640培养基。在培养细胞之前，将青霉素链霉素溶液、胎牛血清与培养基以1∶10∶100的体积比混合，配制成含有血清与双抗的培养基。设置细胞培养箱内的温度为37 ℃，CO_2_含量为5%。细胞传代时加入0.25%的胰蛋白酶进行消化。

### 1.3 CCK8检测分析

将4种不同肿瘤细胞以每孔5×10^4^个/mL的密度加入每个孔中（96孔板），培养12 h后使细胞贴壁，加入不同浓度的h-SPM或探针分子，每个浓度设定5个复孔。将CCK8试剂与培养基以1∶10的体积比混合，配制成含有CCK8试剂的培养基。在药物或探针处理细胞12~48 h后，吸去培养基，每孔加入100 μL含CCK8试剂的培养基继续培养2 h，测定350 nm波长下的吸光度，并根据吸光度计算出药物处理孔的细胞活力，绘制浓度-效应折线图。

### 1.4 荧光凝胶成像

将MDA-MB-231细胞培养在6 cm的培养皿中，当细胞覆盖度达到70%~80%时，加入探针或h-SPM。培养1 h后，吸去培养基，每个培养皿中加入1 mL PBS，去掉皿盖，置于冰上以防止细胞被紫外灯加热。使用350 nm紫外光处理细胞15 min。紫外线处理完毕后，每皿细胞用200 μL的RIPA裂解液进行裂解，超声后离心，去除细胞碎片，获得含有细胞大部分蛋白质的上清溶液。

先配制点击反应所需的相关试剂的混合液：分别量取4 μL含20 mmol/L叠氮罗丹明的DMSO溶液、8 μL含50 mmol/L硫酸铜的水溶液、12 μL含25 mmol/L TCEP的PBS溶液、24 μL含1.67 mmol/L TBTA的DMSO溶液，在1.5 mL的离心管中混合均匀后加入PBS进行稀释。随后向上述试剂的混合液中加入细胞裂解液，再次混合均匀后开始点击化学反应。在常温下将上述反应体系在旋转混合仪上孵育1~2 h，完成点击反应。获得的样品进一步进行聚丙烯酰胺凝胶电泳，将含有蛋白质的凝胶置于荧光凝胶成像系统中，可观察到凝胶中被罗丹明标记的蛋白质条带。

### 1.5 基于活性的蛋白质组分析

进行点击化学反应的过程如1.4节所述，将点击化学反应体系中的叠氮罗丹明换成叠氮生物素，最终获得由生物素标记的蛋白质样品。将这些蛋白质进行纯化，用含0.25% SDS的PBS溶液复溶，加入链霉亲和素磁珠，在4 ℃条件下孵育过夜，吸去上清液，用PBS漂洗磁珠以去除非特异结合的蛋白质后，用胰蛋白酶消化磁珠上富集的蛋白质。将消化后的蛋白质用DTT与IAA进行变性与烷基化处理，之后用固相萃取柱进行除盐操作，低温干燥后用0.1%的TFA溶液溶解，并用质谱仪进行鉴定，测量样品中蛋白质的种类与含量。

色谱条件：C18液相色谱柱（150 mm×150 μm，1.9 μm）；流动相A为0.1% FA水溶液，B相为含有80% ACN和0.1% FA的水溶液；梯度洗脱程序如下：0~2 min，2%B~8%B；2~100 min，8%B~45%B；100~103 min，45%B~95%B；流速设定为500 nL/min。

质谱条件：正离子数据采集依赖（data dependent acquisition，DDA）模式。全扫描范围为*m/z* 350~2 000，质量分辨率为120 000。

### 1.6 潜在作用靶点的生物信息学分析

本研究对筛选获得的差异表达蛋白质进行了系统的生物信息学分析。首先，基于质谱鉴定结果，利用在线工具WEB-based GEne SeT AnaLysis Toolkit （WebGestalt）对目标蛋白质集合进行了基因本体（Gene Ontology， GO）富集分析。通过评估富集显著性，筛选出若干高度富集的信号通路与功能模块。进一步，对这些蛋白质进行功能归类，重点关注其亚细胞定位、功能注释及所涉生命活动的统一性。结合通路分析结果，深入考察显著富集通路中蛋白质的组成特征，识别其共有的定位、功能或调控模式。通过整合上述多层次信息，最终系统地推断h-SPM潜在的作用靶标蛋白质及其可能影响的分子信号通路。

### 1.7 质粒与细胞系的构建

通过相关网站（https：//www.sigmaaldrich.cn/CN/zh/semi-configurators/shrna？term=RB1）等查询潜在靶标蛋白质APP编码基因的shRNA序列，并设计出连接在pLKO.1质粒上的两条互补链序列。pLKO.1质粒上有氨苄青霉素与嘌呤霉素的抗性基因，可用于后续的菌株与细胞系的筛选。设计好的DNA序列由上海生工公司进行合成。将获得的两条互补引物混合后，通过加热后退火的方式使其形成带有黏性末端的双链oligo结构。选用黏性末端对应的限制性内切酶EcoRⅠ与AgeⅠ处理pLKO.1质粒，回收线性化的质粒载体，使用T4连接酶将线性化的pLKO.1质粒与oligo连接，并转化至大肠杆菌DH5α感受态细胞，将转化后的细菌接种至含有氨苄青霉素的LB培养皿中，在37 ℃下培养16 h，筛选阳性克隆，测序确认正确，即完成shRNA载体构建。

用转染试剂（PEI）、包装质粒（psPAX2及pVSV-G）与shRNA载体或空载体pLKO.1（shcon）混合，加入到293T细胞培养基中进行转染。转染12 h后更换一次培养基，继续培养36 h之后收集细胞的培养基，并使用孔径为0.22 μm的滤膜对培养基进行过滤，得到病毒液。将病毒液与培养基以2∶1的体积比混合用于培养MDA-MB-231细胞，12 h后换成新鲜的培养基，再培养36 h之后，向这些细胞系中加入嘌呤霉素，筛选出能够表达出抗性的细胞，筛选7~10天后，提取细胞蛋白质，使用Western Blotting实验验证细胞中对应蛋白质的表达情况。

### 1.8 溶酶体染色

将细胞培养于3 cm的免疫荧光专用培养皿中，培养至覆盖度达到70%~80%后吸去培养基，分别使用浓度为0和10 μmol/L的h-SPM处理细胞，并在培养1、3、6 h后吸去培养基。将Lysotracker以1∶2 000的体积比加入到无血清的培养基中，配制成含有染料的培养基，将该培养基与细胞共孵育2 h以完成染色。染色完毕使用4%的多聚甲醛固定细胞，将细胞进行共聚焦成像，观察细胞的染色情况并拍照，之后将照片使用Image J软件处理，得到溶酶体染色的定量数据，并绘制出柱状图。

### 1.9 Western Blotting实验

吸去培养皿中的培养基，将RIPA裂解液加入到培养皿中，用细胞铲刮下细胞，并用移液枪吸取裂解液转移至1.5 mL的离心管中，将裂解液置于冰上。用超声仪对裂解液进行超声，每次5~10 s，一共3次。超声完毕后进行离心，去除细胞碎片，吸取上清液。用BCA试剂检测上清液的蛋白质浓度，并用RIPA裂解液进行调平，使各样品的蛋白质浓度相同。加入含有SDS与溴酚蓝，且能够使蛋白质变性的蛋白上样缓冲液后，在98 ℃金属浴中加热10 min使样品中的蛋白质充分变性，使用10%的聚丙烯酰胺凝胶进行电泳。

电泳完成后，将聚丙烯酰胺凝胶中的蛋白质转移至PVDF膜上，用质量分数为5%的脱脂牛奶对PVDF膜进行封闭，在4 ℃的条件下用特异性一抗孵育PVDF膜过夜，清洗3次，并根据一抗来源用二抗孵育PVDF膜，清洗后用发光液孵育PVDF膜，在化学发光成像系统上进行蛋白质条带的成像，进而测定相关蛋白质在细胞中的相对含量。

### 1.10 数据处理与统计分析

所有实验数据均以3次重复实验的平均值±标准差（standard deviation， SD）表示。使用Microsoft Excel和 GraphPad Prism 5绘制图表。两组间比较采用*t*检验，多组间比较采用单因素方差分析。*p*值小于0.05表示差异具有统计学意义。

## 2 结果与讨论

### 2.1 h-SPM及其探针分子的抗肿瘤活性评价

首先，基于h-SPM的结构特征设计并合成了与其结构相似、并在分子中引入炔基及二氮丙啶基团的探针分子（图1）。为了验证探针分子与h-SPM具有类似的细胞毒性，本研究选取了4种肿瘤细胞系（MDA-MB-231、LN229、A549和MHCC-97H）进行评价。采用不同浓度（0、0.8、1.6、3.2、6.3、12.5、25、50、100 μmol/L）的h-SPM及探针分别处理细胞，通过细胞增殖抑制实验分析其活性。结果如图2所示，h-SPM对所选肿瘤细胞系均表现出显著的抑制活性，而探针分子的剂量-效应曲线与h-SPM高度相近，表明其细胞毒性与h-SPM相近，所加入的功能性基团未显著改变h-SPM的生物学活性。因此，该探针可用于后续针对h-SPM作用靶点和机制的研究。

**图1:**
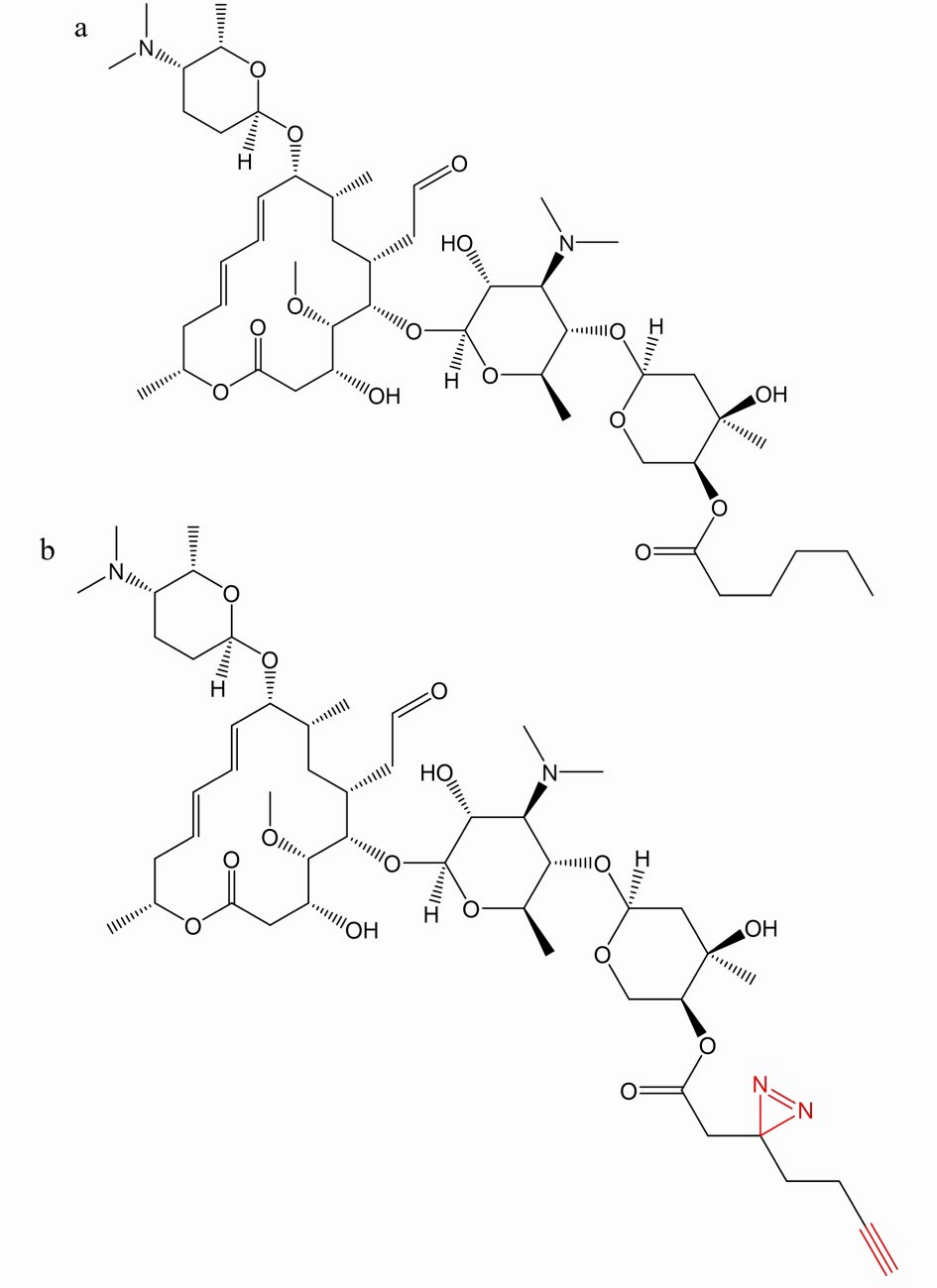
（a）药物h-SPM与（b）探针的化学结构

**图2:**
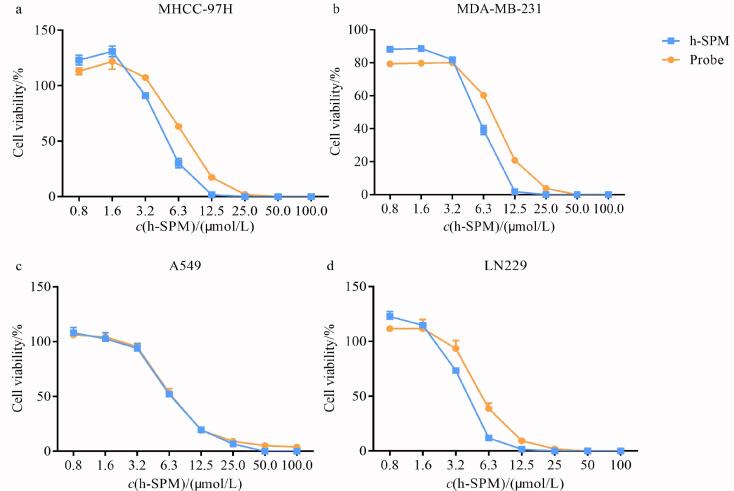
h-SPM与探针的细胞增殖抑制实验（*n*=3）

### 2.2 ABPP-荧光凝胶成像分析

本研究通过ABPP-荧光凝胶成像技术进行分析。实验共设置9个处理组：第1、2组加入5 μmol/L的探针；第3、4、5组分别加入1、5、10 μmol/L的探针；第6、7、8、9组均加入5 μmol/L探针，并分别同时加入0、5、10、25 μmol/L的h-SPM；其中第1组不进行紫外线处理。凝胶内荧光成像结果如图3所示。

**图3:**
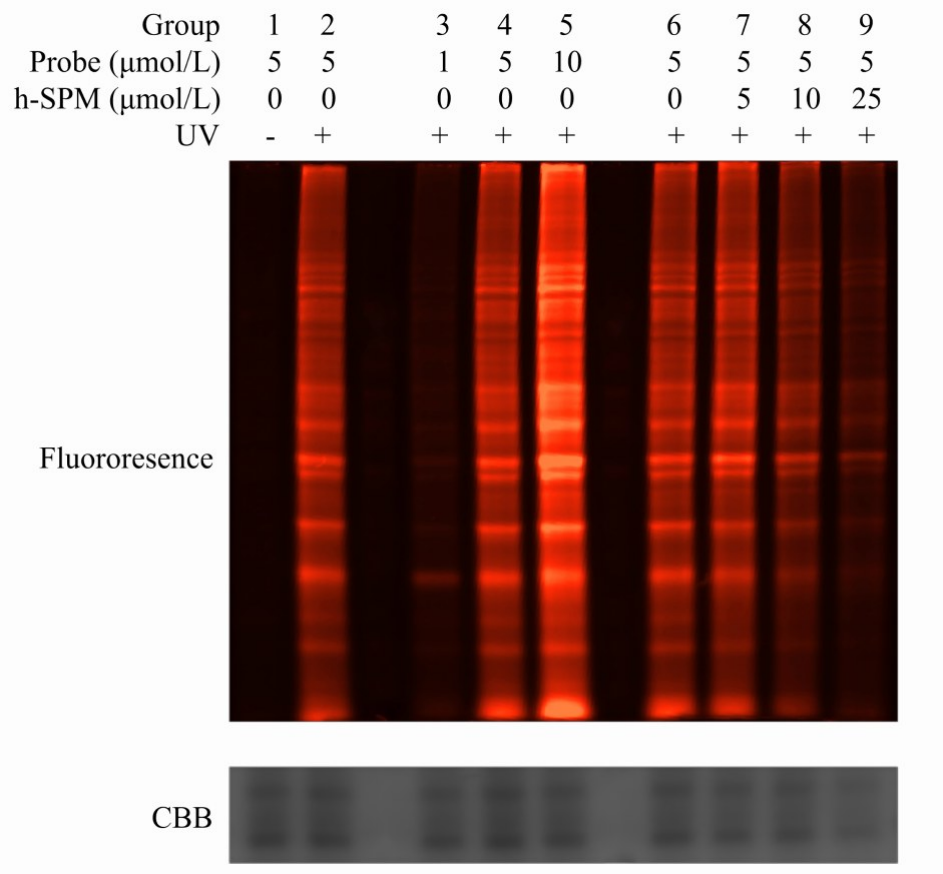
探针标记蛋白质的荧光凝胶成像

从图3可以看出，未经紫外线照射的第1组样品几乎未检测到荧光条带，而经照射的第2组样品则呈现清晰的条带信号，表明紫外线照射有效促进了探针与蛋白质之间的光交联反应，使探针通过点击反应成功标记上罗丹明荧光基团。第1组样品中可能存在的微弱非共价结合，在蛋白质提取过程中发生解离，进一步证实若未形成共价连接，则无法通过点击反应实现稳定标记。该结果表明紫外线处理是探针与靶点蛋白质进行共价结合并完成后续荧光标记的关键步骤。

在不同探针浓度（1、5、10 μmol/L）处理的第3、4、5组样品中，荧光条带信号强度随着探针浓度升高而增强，呈现剂量依赖性关系。这表明在本实验条件中，探针浓度尚未达到结合饱和，从而可避免因探针浓度过大导致的非特异性标记增强，有利于减少假阳性结果。

在相同探针浓度（5 μmol/L）的第6~9组样品中，随着共孵育的h-SPM浓度（0、5、10、25 μmol/L）增加，荧光条带信号逐渐减弱，提示h-SPM与探针在结合蛋白质时存在竞争关系，进一步印证探针与药物在结构和结合特性上具有高度相似性。此竞争抑制现象也为后续基于质谱的定量分析提供了方法学依据。

### 2.3 ABPP-质谱鉴定及潜在靶点验证分析

为鉴定h-SPM的潜在作用靶点，本研究采用竞争性ABPP策略结合定量蛋白质组学技术进行分析。设立两组样品：实验组单独加入浓度为5 μmol/L的探针；竞争组在加入5 μmol/L探针的同时共孵育25 μmol/L h-SPM。通过质谱检测并比较两组中蛋白质的丰度，计算各蛋白质在实验组与竞争组中的信号强度比值，该比值越高，表明该蛋白质与探针的结合受到h-SPM的竞争抑制越显著，提示其作为h-SPM直接作用靶点的可能性越大，通过设置比值阈值，舍去比值过低的蛋白质即可进行筛选^［15，16］^。本研究参考了现有的基于ABPP技术的药物靶点发现策略，为确保GO富集分析的可靠性，需将目标蛋白质集合控制在适宜规模。因此，将比值≥1.5作为筛选阈值，从质谱鉴定的1 317种蛋白质中，筛选得到184种潜在靶点蛋白质，约占鉴定蛋白质总数的14%。该蛋白质集合被用于后续的生物信息学分析。

对筛选获得的候选蛋白质进行GO通路富集分析，结果如表1所示。分析显示，这些蛋白质显著富集于炎症反应、囊泡转运与细胞内吞作用等相关通路。此外，在筛选得到的众多蛋白质中，存在多个定位于细胞膜或溶酶体膜的蛋白质，提示h-SPM可能通过胞吞等转运途径进入细胞，并与这些参与膜转运的蛋白质分子发生相互作用。

**表1:** GO分析结果相关的细胞通路

Gene set	Description
GO：0016192	vesicle-mediated transport
GO：0045321	leukocyte activation
GO：0045055	regulated exocytosis
GO：0002263	cell activation involved in immune response
GO：0002274	myeloid leukocyte activation
GO：0001775	cell activation
GO：0043312	neutrophil degranulation
GO：0002283	neutrophil activation involved in immune response
GO：0042119	neutrophil activation
GO：0002446	neutrophil mediated immunity

从候选蛋白质中进一步筛选获得定位于细胞膜、囊泡膜与溶酶体膜表面的蛋白质，包括APP、LDLR、TFRC与IGF2R 4种蛋白质^［17-20］^。如图4所示，通过Western Blotting检测，比较空白对照组与10 μmol/L h-SPM处理条件下上述蛋白质表达水平的变化，结果显示，在h-SPM处理3 h后，APP与LDLR的蛋白质表达水平显著下调，将药物处理时间延长到6 h，蛋白质水平下调的现象更加明显。这一现象提示APP与LDLR很可能参与h-SPM所诱导的生物学过程活动中，其表达下调可能与药物直接结合并影响蛋白质稳定性有关，亦可能通过其他间接调控机制实现。综上所述，h-SPM处理可显著下调蛋白质APP与LDLR的表达水平，说明二者的表达受h-SPM调控，由此推测APP与LDLR可能是h-SPM的潜在作用靶点。

**图4:**
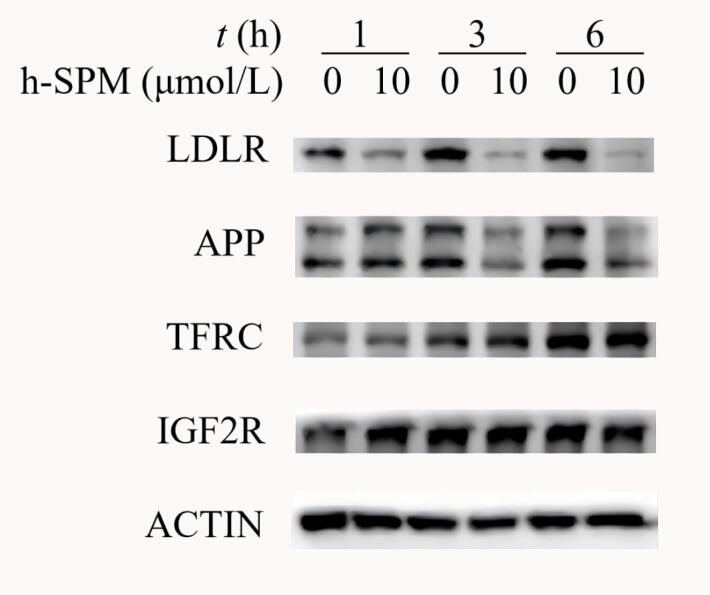
潜在靶点蛋白质的免疫印记实验

### 2.4 潜在靶蛋白质APP对正己酰螺旋霉素敏感性的验证

为进一步验证候选靶点蛋白质是否参与h-SPM的作用机制，本研究采用shRNA介导的基因敲低技术进行分析。鉴于候选靶点蛋白质APP与细胞的凋亡相关^［21］^，蛋白质APP被选择作为代表性溶酶体相关蛋白质，评估其对h-SPM的敏感性。首先，在MDA-MB-231细胞中使用特异性shRNA成功构建了APP稳定敲低的细胞系（图5a）。随后将APP敲低细胞及对照细胞制备成1×10^5^个/mL的细胞悬液，按每孔100 μL培养基（即1×10^4^个细胞）接种至96孔板中，培养12 h使细胞充分贴壁后，更换为含有不同浓度h-SPM（0、0.8、1.6、3.2、6.3、12.5、25、50、100 μmol/L）的培养基继续处理。每个药物浓度设5个复孔，培养12 h后弃去培养基，采用CCK8试剂检测细胞活力，并绘制细胞活力与药物浓度的关系曲线。如图5b所示，在h-SPM浓度为12.5 μmol/L时，3种APP敲低细胞系的细胞活力均显著高于对照组细胞（*p*<0.05），表明当蛋白质APP水平下调时，细胞对h-SPM的敏感度明显降低，提示蛋白质APP在h-SPM介导的细胞增殖抑制作用过程中具有关键功能。

**图5:**
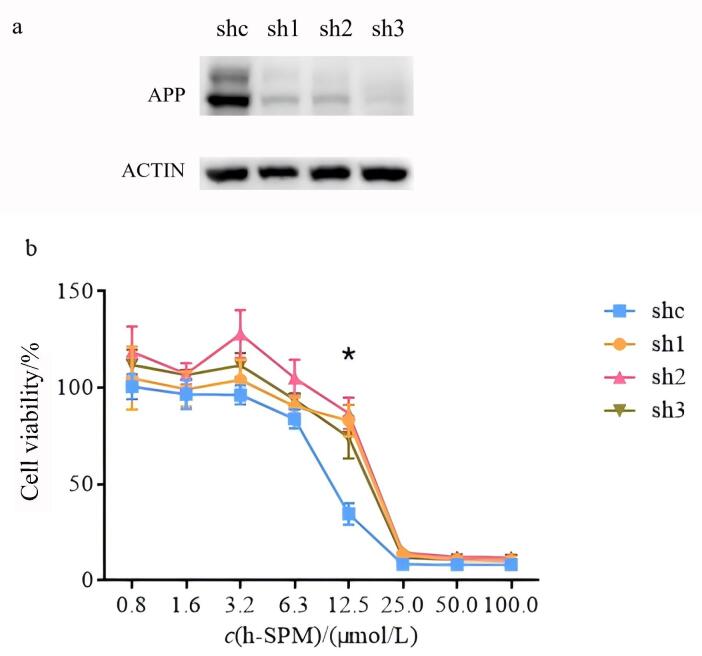
shRNA敲低蛋白质APP的细胞系增殖抑制实验（*n*=3）

### 2.5 溶酶体染色分析

APP定位于溶酶体，而h-SPM靶向作用于APP，提示h-SPM可能主要作用于溶酶体。基于蛋白质组学的生物信息学分析结果显示，内吞调控相关通路是显著富集的通路之一，且该通路中多数蛋白质定位于溶酶体相关膜结构上，提示溶酶体在h-SPM作用于细胞的过程中具有重要作用，因此通过检测药物处理后溶酶体的变化来验证这一猜想。

采用溶酶体特异性荧光探针Lysotracker对溶酶体进行染色^［22，23］^，通过检测荧光信号强度反映溶酶体状态。在h-SPM处理不同时间后，对荧光强度进行定量分析，结果如图6a所示。与对照组相比，h-SPM处理3 h及6 h后，溶酶体荧光信号强度显著降低。进一步通过Western Blotting实验检测细胞中溶酶体标志蛋白质LAMP1以及CTSD的表达水平，发现随着药物处理时间延长，这两种蛋白质的表达水平均呈下降趋势（图6b）。上述结果表明，h-SPM可影响溶酶体的稳定性或数量，并可能通过破坏溶酶体功能干扰细胞正常生理活动。

**图6:**
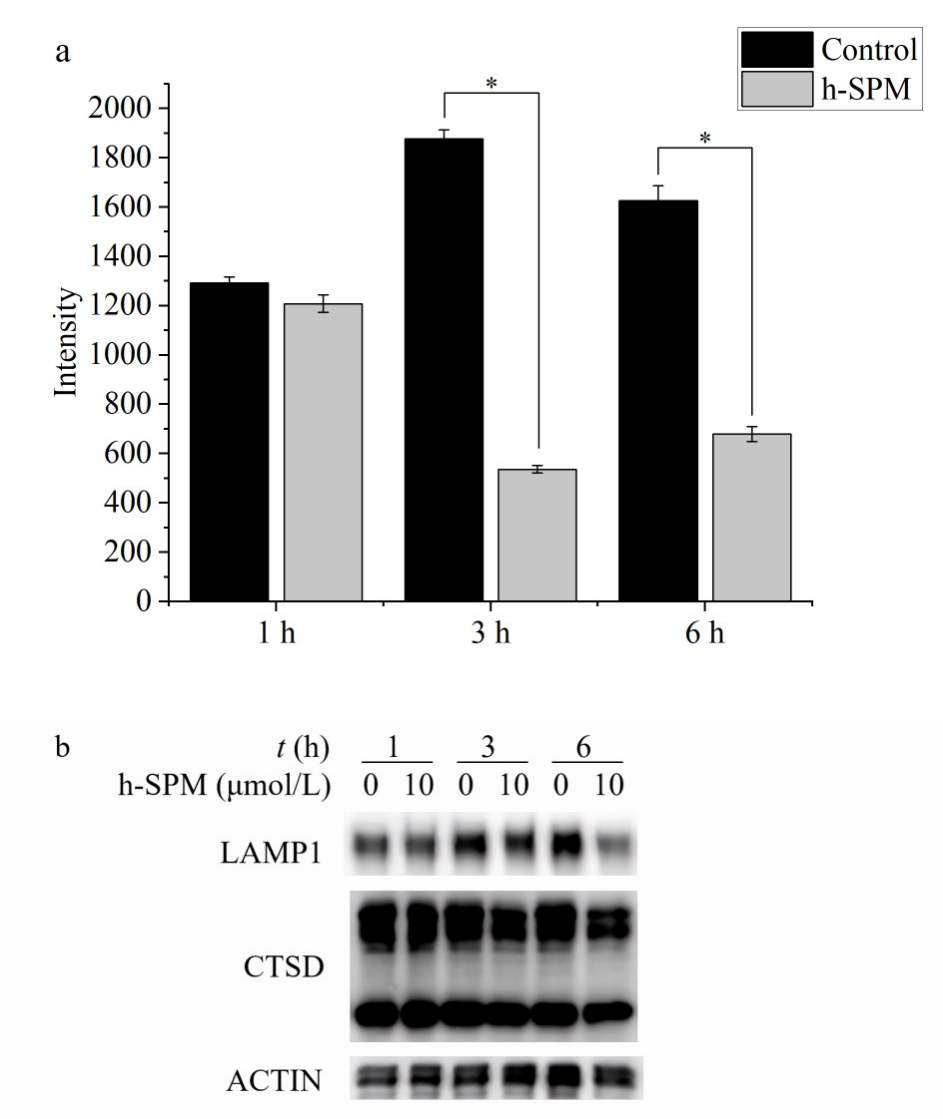
药物对细胞溶酶体的影响

## 3 结论

本研究综合运用ABPP、质谱检测、GO富集分析及shRNA介导的基因敲低等方法，系统地探索了药物h-SPM的分子作用机制。通过ABPP富集并结合质谱鉴定分析，提示蛋白质APP可能作为h-SPM的潜在作用靶点；GO分析进一步显示药物处理与溶酶体功能及内吞途径密切相关。后续功能验证实验表明，通过shRNA敲低APP的细胞对药物的敏感度显著降低，证实APP在h-SPM作用于细胞过程中发挥关键作用。同时，溶酶体染色及溶酶体标志蛋白质检测结果显示，h-SPM处理细胞后，可导致溶酶体数量减少及相关蛋白质表达下调。综合以上结果进行推测，h-SPM可能通过与APP等溶酶体相关膜蛋白质结合进入溶酶体并在其中积累，而该药物结构中存在氨基这种碱性基团，能够结合溶酶体中的氢离子，影响溶酶体内部的酸性环境，干扰其pH稳态，进而导致溶酶体的功能紊乱、结构破坏，最终诱导细胞死亡。为了更全面地理解h-SPM与蛋白质APP的靶向作用，阐明h-SPM抗肿瘤作用靶点与机制，后续APP等靶点将在更多肿瘤细胞及体内动物模型中进行实验，以验证在h-SPM介导的溶酶体功能障碍及抗肿瘤效应中的普遍性。

综上所述，本研究发现h-SPM加入细胞以后，主要靶向溶酶体相关结构，其作用机制可能涉及与APP、LDLR等溶酶体相关蛋白的相互作用，干扰这些蛋白在细胞膜与溶酶体膜之间的流动，进而引起溶酶体结构损伤与数量减少等现象，这可能是h-SPM发挥抗肿瘤活性的重要分子机制之一。这项研究一方面为螺旋霉素类药物的“老药新用”提供了新的理论依据和潜在的分子标志物（如APP表达水平），有助于筛选可能从该疗法中获益的患者群体；另一方面，揭示了靶向溶酶体稳态可作为肿瘤治疗的一个新策略，为开发新型溶酶体靶向抗肿瘤药物提供了思路。
